# The association between household bed net ownership and all-cause child mortality in Madagascar

**DOI:** 10.1186/s12936-016-1520-2

**Published:** 2016-09-17

**Authors:** Dominique Meekers, Joshua O. Yukich

**Affiliations:** 1 Department of Global Community Health and Behavioral Science, Tulane University School of Public Health and Tropical Medicine, New Orleans, LA USA; 2 Department of Tropical Medicine, Center for Applied Malaria Research and Evaluation, Tulane University School of Public Health and Tropical Medicine, New Orleans, LA USA

**Keywords:** Malaria, Bednets, Child mortality, Madagascar, Plasmodium

## Abstract

**Background:**

Malaria continues to be an important cause of morbidity and mortality in Madagascar. It has been estimated that the malaria burden costs Madagascar over $52 million annually in terms of treatment costs, lost productivity and prevention expenses. One of the key malaria prevention strategies of the Government of Madagascar consists of large-scale mass distribution campaigns of long-lasting insecticide-treated bed nets (LLIN). Although there is ample evidence that child mortality has decreased in Madagascar, it is unclear whether increases in LLIN ownership have contributed to this decline. This study analyses multiple recent cross-sectional survey data sets to examine the association between household bed net ownership and all-cause child mortality.

**Results:**

Data on household-level bed net ownership confirm that the percentage of households that own one or more bed nets increased substantially following the 2009 and 2010 mass LLIN distribution campaigns. Additionally, all-cause child mortality in Madagascar has declined during the period 2008–2013. Bed net ownership was associated with a 22 % reduction in the all-cause child mortality hazard in Madagascar.

**Conclusions:**

Mass bed net distributions contributed strongly to the overall decline in child mortality in Madagascar during the period 2008–2013. However, the decline was not solely attributable to increases in bed net coverage, and nets alone were not able to eliminate most of the child mortality hazard across the island.

## Background

Madagascar has achieved substantial success in reducing the infant and child mortality rate. Data from the Demographic and Health Surveys and the Millennium Development Survey show that the infant mortality rate (_1_q_0_) had decreased from 93 per 1000 live births for the period from 1989–93 to 48 for the period between 2004/5 and 2008/9, and further to 42 for the 5-year period before the 2012/13 survey. Similarly, the under-five mortality rate (_5_q_0_) has decreased from 163 to 72 per 1000 live births, and further to 62 [1, 2]. Despite this impressive progress, Madagascar still suffers from several major health problems, including malaria.

Because of large differences in climate and altitude in Madagascar, not all parts of the island are equally affected by malaria [3–7]. Based on the duration and risk of transmission, four major epidemiological zones can be identified: (1) an equatorial zone located in the Eastern part of the country where transmission is high and perennial; (2) a tropical zone in the Western part of the country which has a long transmission period of more than 6 months; (3) a sub-desert zone with short episodes of transmission, and (4) the elevated highlands where there is only short seasonal transmission of malaria [8]. Although the transmission period varies by zone, it is highest between October and April, when it tends to be rainy and hot [9].

Madagascar has had a formal malaria policy since 1998. The policy was updated in 2005 and further revised in 2012. The Government of Madagascar ratified the declaration on the roll back malaria initiative in 2000, which aimed to achieve—among other objectives—that 60 % of children under the age of 5 would benefit from preventive measures, such as bed nets. The 2008–12 strategic plan aims to ensure universal access to prevention, diagnosis, and treatment to reduce the impact of and eventually eliminate malaria. Because of the existence of different transmission zones, appropriate strategies to combat malaria vary by region. However, one of the long-term objectives of the strategic plan is to achieve that 100 % of households possess at least two LLIN [8, 10]. Consequently, mass LLIN distribution campaigns have—in different phases—targeted most of the country, with the exception of the central upper highlands [9, 10].

To supplement routine ITN/LLIN distribution and social marketing of bed nets, mass distribution campaigns took place in 2007 (1.8 million nets), 2009 (1.6 million), 2010 (5.7 million), and 2012 (3.6 million). The 2007 integrated measles/malaria campaign was implemented as part of the semi-annual mother and child health week in October 2007 and targeted the West coast, South and North [11, 12]. The 2009 campaign targeted 19 districts located in four administrative regions in the Eastern part of the country, which are characterized by high and perennial malaria transmission (Atsinanana, Votavavy Fitovinany, Atismo Atsinanana, and Anosy), while the 2010 campaign targeted the remaining regions. The 2012 distribution campaign targeted the same regions as the 2009 campaign, but adding Sava and Analanjirofo [13–16].

According to the 2008/9 Demographic and Health Survey and 2011 and 2013 Malaria Indicator Surveys, the percentage of households that owned at least one bed net increased from 57 % in 2008/9 to 83 % in 2011, but subsequently declined to 72 % in 2013. Similarly, the percentage of children under the age of 5 who slept under an ITN on the night prior to the survey increased from 46 % in 2008/9 to 79 % in 2011, but declined to 65 % in 2013 [9, 10, 17].

## Methods

### Data sources

This report analyses data from four different surveys: the 2008/9 Madagascar Demographic and Health Survey (DHS), the 2011 Madagascar Malaria Indicator Survey (MIS), the 2012/13 Madagascar Millennium Development Goals Survey (MDG),^1^ and the 2013 Madagascar Malaria Indicator Survey [8–10, 17]. Each of the surveys used a two-stage stratified random sample to select a representative sample of households. A household questionnaire was administered to collect information about the household members, the household’s socioeconomic status, bed nets available in the household, and other factors. In the selected households, all women aged 15–49 were also interviewed.

The 2008/9 DHS and 2012/13 MDG surveys both aimed to collect information on a wide range of health topics. The 2008/9 DHS contains data on a nationally representative sample of 17,957 households and on 17,375 women aged 15–49. The 2012/13 MDG sample includes 19,488 households and 15,675 women aged 15–49. The objective of the 2011 and 2013 MIS surveys was to collect data on malaria prevention and control. These surveys are representative only of those regions where malaria is either endemic or epidemic. The 2011 MIS survey includes data on 8094 households and 8169 women aged 15–49. The 2013 MIS sample includes 8575 households and 8045 women aged 15–49.

Because the MIS surveys were restricted to zones where malaria is endemic or epidemic, communities located at an altitude above 1500 m were excluded from the MIS sampling frame, as there is little or no malaria transmission at such high altitudes. Because there is no malaria transmission in Antananarivo (the capital), Antsirabe I, and Fianarantsoa I, other than imported cases, these cities were also excluded [9, 10]. In practice, these exclusions imply that large parts of Vakinankaratra as well as smaller parts of other regions were omitted from the sampling frame for the MIS surveys. As such, the MIS data are not fully comparable with those from the DHS and MDG surveys. Because the DHS and MDG surveys do not contain a variable that enables us to identify the zones where malaria is endemic or epidemic, it is not possible to fully correct for these sampling differences. However, to increase the comparability of the samples, all clusters in the 2008/9 DHS that were located above 1500 m were excluded.^2^ In addition, the capital, Antananarivo, was removed from the 2008/9 DHS and 2012/13 MDG samples. This reduces the number of households in the working samples to 15,675 for the DHS, 15,588 for the MDG, 8094 for the 2011 MIS, and to 8.575 for the 2013 MIS survey.

To further minimize the effect of these sampling differences, all data are reported separately for each of Madagascar’s 22 major administrative regions (*faritra*). The key characteristics of the resulting working samples are shown in Table 1. The results show that the regional distribution of households in the four surveys is comparable, with some minor exceptions that are due to the sampling differences described earlier. The most notable differences are the relatively high proportion of households from Vakinankaratra in the 2012/13 MDG survey (8.3 %), from Vatovavy Fitovinany in the 2013 MIS (9.0 %) and from Atsimo Andrefana in the 2011 MIS (8.9 %).

**Table 1:** Sample characteristics

	Survey			
	2008–9 DHS	2011 MIS	2012–13 MDG	2013 MIS
Survey features				
Nationwide	Nationwide	Malaria areas	Nationwide	Malaria areas
Mortality data	Yes	No	Yes	No
Altitude data	Yes	Yes	No	Yes
Number of households	15,675	8094	15,588	8575
Percentage distribution of households				
Antananarivo				
Analamanga	11.7	10.8	10.5	11.2
Vakinankaratra	4.0	2.7	8.3	1.8
Itasy	3.7	3.5	3.3	2.4
Bongolava	3.0	2.4	2.1	3.2
Fianarantsoa				
Haute Matsiatra	5.3	5.1	5.1	5.4
Amoron i Mania	3.7	3.3	3.1	3.1
Vatovavy Fitovinany	5.4	5.1	6.4	9.0
Ihorombe	2.1	2.4	1.4	.9
Atsimo Atsinanana	3.2	3.9	3.6	4.6
Toamasina				
Atsinanana	6.4	6.3	7.3	6.4
Analanjirofo	7.1	4.3	6.1	5.6
Alaotra Mangoro	6.3	4.8	5.2	7.3
Mahajanga				
Boeny	3.9	5.7	4.1	3.8
Sofia	6.4	6.0	6.2	7.0
Bestiboka	1.5	1.9	1.4	1.3
Melaky	1.2	1.4	1.4	2.5
Toliary				
Atsimo Andrefana	6.5	8.9	6.5	6.2
Androy	3.1	3.6	3.0	3.6
Anosy	3.3	3.4	3.2	2.9
Menabe	2.9	3.4	2.8	3.0
Antsiranana				
Diana	3.4	4.5	4.1	4.5
Sava	6.0	6.8	5.2	4.2
	100 %	100 %	100 %	100 %

For the analyses, a merged file was created that contains relevant data for all the households included in the four surveys. In addition, based on data from the birth histories in the four surveys, a merged child file was created that contains data for each of the children reported in the birth histories. The latter data file includes not only survivorship data, but also selected data derived from the household questionnaire (e.g., whether the child lives in a household that owns at least one or two bed nets, etc.).

### Measures

Ideally, a study on the association between ITN availability and child mortality would focus on malaria-related child deaths only. Unfortunately, collecting cause of death data in household-based surveys is nearly impossible, particularly retroactively. The 2008/9 DHS and 2012/13 MDG surveys contain complete birth histories that include the date of birth and date of death for deceased children, but as anticipated information on their cause of death is not available. Hence, it is only possible to calculate mortality from all causes combined. The indicators of all-cause mortality are the probability of surviving to age 5 (_5_p_0_) and the probability of surviving to age 1(_1_p_0_), which are estimated using life table (survival) analyses [18]. Since estimating these survival probabilities requires information on age at death, they can only be calculated for the 2008/9 DHS and 2012/13 MDG surveys.^3^

The indicators of net ownership are based on data from the household questionnaires. All four surveys collected information on the number of bed nets in the household. The indicators of bed net ownership are a dichotomous variable that indicates whether or not a household possesses at least one ITN and a similar dichotomous variable that indicates whether the household possesses at least two ITNs.

### Analyses

The analysis consists of three main parts: (1) a descriptive analysis of trends in infant and child mortality, (2) a descriptive analysis of trends in bed net ownership, and (3) a multivariate analysis of the association between bed net ownership and infant/child mortality.

The aim of the analysis of trends in infant and child mortality is to describe any secular trends in mortality that existed before the mass ITN distribution campaigns started. To do this, life table analyses was used to calculate the probability of surviving to age 1 and the age of 5, by birth cohort [19]. Results are presented for the total sample, as well as by administrative region, and type of place of residence (rural/urban).

Next, the analysis examines trends in the percentage of households that report possessing at least one bed net and the percentage that report possessing at least two nets. Because these data refer to the number of bed nets available in the household at the time of the survey, trends are shown by survey wave. Again, results are shown for the total survey, as well as separate results by administrative region and by rural/urban residence.

Finally, Cox regression was used to estimate the association between bed net ownership and the risk of dying before age 1 and before the age of 5, after controlling for other factors [20–22]. To do this, a survival time data file was created in which each child is represented by multiple observations. Specifically, observations are available for each month a child is at risk of dying, starting with the month of birth through age 1 or 5 (for infant/child mortality) or death, whichever came first. The analysis is limited to children born during the 5 years preceding the DHS and MDG surveys. As such, the data include a total of 22,792 children, who represent nearly 653,000 person-months of risk for the child mortality analysis and 234,000 person-months of risk for the infant mortality analysis. As before, the key programme exposure variables are two dichotomous variables indicating whether or not the child lives in a household that possesses one or more, or two or more bed nets.

To account for the secular trend in infant and child mortality, the analyses include the calendar year of observation as a predictor (coded as 2003–08, 2009, 2010, 2011, or 2012/13). It is anticipated that the declining secular trend in infant and child mortality that has been documented in the literature will also be observed in the present analysis. The mass ITN distribution campaigns that started in 2009 may accelerate this decline, but most likely only with a lag of at least 1 year (i.e., the effect of the 2009 distribution campaign is unlikely to be observed before 2010).

**Fig. 1:**
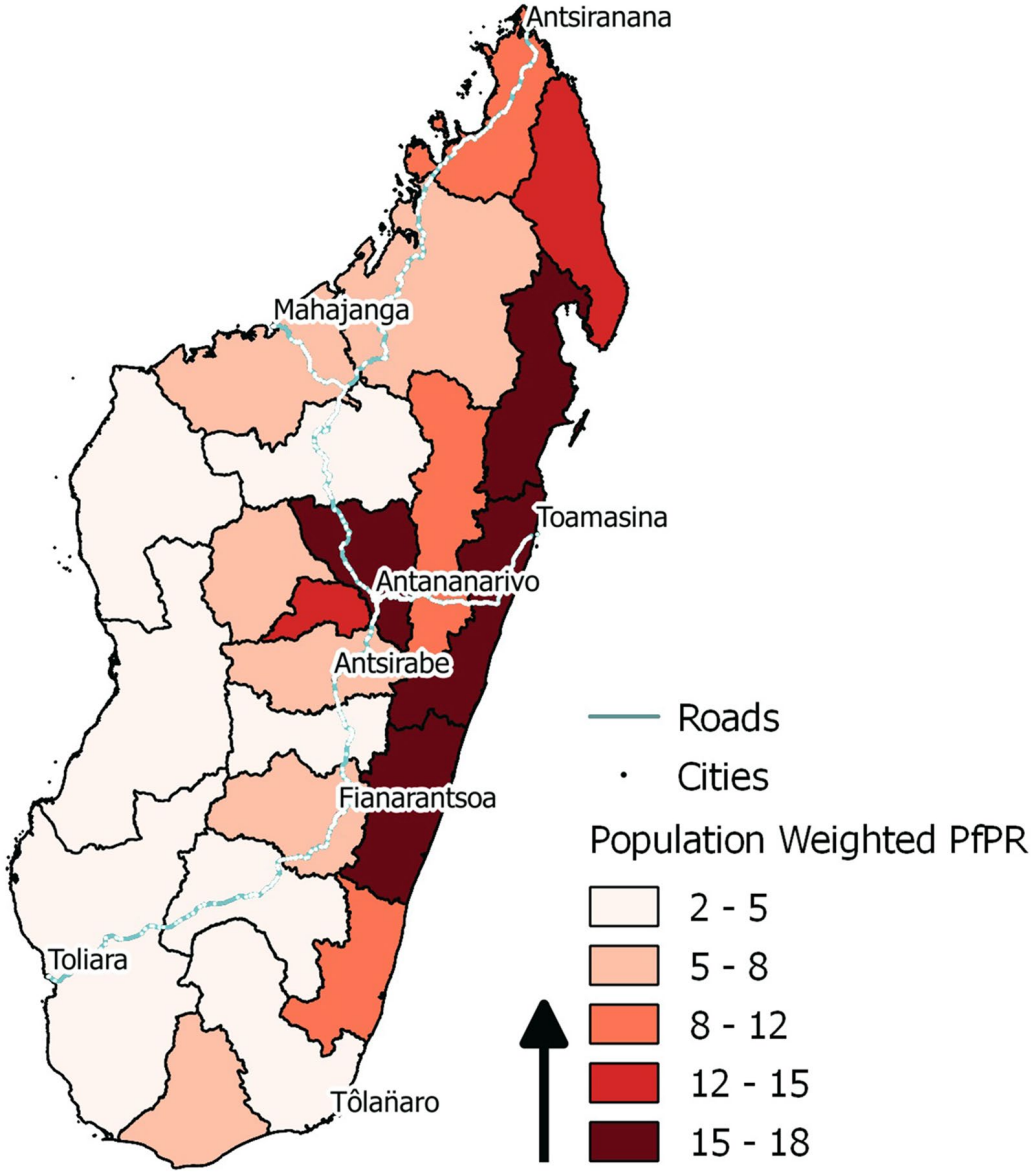
Regional *Pf*PR_2–10_ summarized as mean per region after weighting *Pf*PR_2–10_ estimates by local population densities (*darker red* indicates regions with higher population weighted mean *Pf*PR_2–10_)

To account for differences in the malaria transmission intensity, a categorical variable was created that equals the mean *Plasmodium falciparum* parasite rate for children aged 2–10 (*Pf*PR_2–10_) for each of the 22 administrative districts [23] after weighting by population density using the global rural urban mapping project dataset for Madagascar (see Fig. 1). *Plasmodium falciparum* accounts for an estimated 85–90 % of all malaria cases in Madagascar [15, 24]. The *Pf*PR_2–10_ estimates are derived from the 2010 data of the Malaria Atlas Project. In addition, a time-varying variable was created that equals one during those months that occurred during the malaria season, and zero otherwise. Because of the diversity in epidemiology, separate estimates of the length of the malaria season were calculated for each administrative region based on MARA/ARMA maps of the first and last months of the malaria season [5, 6]. It is hypothesized that living in a region with a higher *Pf*PR_2–10_ will be associated with higher mortality levels, and that children will experience a higher likelihood of dying during those months that represent the malaria season in their region.

At the household and community level, the analysis controls for the child’s mother’s age, parity, and level of education (none, primary, secondary, or higher), for the wealth quintile of the parental household, and rural/urban residence. Additional controls include the gender of the child, the average diarrhoea prevalence and DPT3 prevalence for the cluster (primary sampling unit) in which the child lives. DPT3 immunizations help prevent diphtheria, whooping cough (pertussis) and tetanus, and the percentage of children who received all three required doses is considered an important indicator of the level of child immunizations [25]. All analyses were conducted in STATA; geographic processing was conducted using QGIS.

## Results

### Trends in infant and child mortality

Table 2 shows the results of life table estimates of the probability of dying before reaching exact age 5 per 1000 live births, by year of birth. Overall, the probability of dying before the age of 5 declined from 95 deaths per 1000 live births for children born prior to 2005, to 61 deaths for those born in 2005/06, and further to 58 deaths for children born from 2007 onward. Breakdown by type of place of residence confirms that a similar pattern of a rapid decline in child mortality followed by near-stagnant mortality levels occurred in both rural and urban areas.

**Table 2:** Life table estimates of the probability of dying before exact age 5 per 1000 live births (_5_q_0_), by birth cohort, region, and type of place of residence (Source: 2008/9 MDHS; 2012–13 ENSOMD)

	Year of birth		
	<2005	2005/06	2007/11
Antananarivo			
Analamanga	67	53	31
Vakinankaratra	78	56	53
Itasy	87	69	49
Bongolava	64	64	33
Fianarantsoa			
Haute Matsiatra	94	55	65
Amoron i Mania	88	39	40
Vatovavy Fitovinany	174	99	87
Ihorombe	96	73	39
Atsimo Atsinanana	140	58	46
Toamasina			
Atsinanana	71	60	64
Analanjirofo	87	37	83
Alaotra Mangoro	94	63	38
Mahajanga			
Boeny	93	67	42
Sofia	72	37	71
Betsiboka	108	96	101
Melaky	85	33	51
Toliary			
Atsimo Andrefana	103	57	57
Androy	82	78	65
Anosy	126	100	66
Menabe	106	50	76
Antsiranana			
Diana	56	27	39
Sava	82	55	64
Residence			
Rural	98	65	60
Urban	79	40	37
Total	95	61	58

Breakdown by administrative region shows that substantial declines in child mortality between the pre-2005 birth cohorts and the 2005/06 cohort were observed in nearly all of the 22 regions, the sole exception being Bongolava where there was no change in the child mortality level. However, from 2007 onward the decline in child mortality clearly slowed down: the probability of dying before the age of 5 declines from 61 per thousand live births for those born in 2005/06 to only 58 per thousand for those born in 2007/11. Examination of the regional variation shows that while child mortality declined in a number of regions, it is starting to increase in regions such as the northern regions of Diana (from 27 to 39 deaths per 1000 live births), and Sava (55 to 64 deaths), and Sofia (37 to 71 deaths). Similar increases are observed in Haute Matsiatra (from 55 to 65 deaths per 1000 live births), Analanjirofo (37 to 83 deaths), Melaky (33 to 51 deaths), Menabe (50 to 76 deaths).

It is noteworthy that three of the four Eastern regions targeted by the 2009 mass ITN distribution campaign (Vatovavy Fitovanany, Atsimo Atsinanana, and Anosy) had exceptionally high levels of child mortality among children born prior to 2005 (174, 140, and 126 deaths per 1000 live births, compared to only 95 deaths for the total sample), but subsequent cohorts experienced major declines in child mortality. The fourth region targeted by the 2009 campaign (Atsinanana) had below average child mortality among children born before 2005, and achieved only very modest improvements.

Trends in infant mortality are shown in Table 3. Because infant mortality requires only a 1-year observation period, it is possible to distinguish between infants born in 2007/08 and those born in 2009/11. As was the case with child mortality, it is observed that infant mortality declined rapidly from 60 deaths per 1000 live births for infants born before 2005 to 42 deaths for those born in 2005 or 2006, but then stagnated reaching 43 deaths for infants born in 2007/08. The data suggest, however, that infant mortality is declining again, dropping to 34 deaths per 1000 births for the 2009/11 birth cohort.

**Table 3:** Life table estimates of the probability of dying before exact age 1 per 1000 live births (_1_q_0_), by birth cohort, region, and type of place of residence (Source: 2008/9 MDHS; 2012–13 ENSOMD)

	Year of birth			
	<2005	2005/06	2007/08	2009/11
Antananarivo				
Analamanga	43	37	29	26
Vakinankaratra	52	35	35	23
Itasy	52	50	53	24
Bongolava	43	47	45	13
Fianarantsoa				
Haute Matsiatra	62	47	68	40
Amoron i Mania	54	34	34	39
Vatovavy Fitovinany	99	47	64	47
Ihorombe	59	51	35	30
Atsimo Atsinanana	85	32	28	23
Toamasina				
Atsinanana	46	30	58	30
Analanjirofo	50	24	42	43
Alaotra Mangoro	53	55	37	35
Mahajanga				
Boeny	59	49	26	22
Sofia	42	27	39	63
Betsiboka	68	55	64	61
Melaky	54	25	31	26
Toliary				
Atsimo Andrefana	75	50	32	51
Androy	58	46	41	41
Anosy	79	63	69	26
Menabe	72	38	38	30
Antsiranana				
Diana	39	23	44	27
Sava	53	37	27	42
Residence				
Rural	62	44	45	35
Urban	49	27	33	33
Total	60	42	43	34

Cohort contains fewer than 100 children

Since most children live in rural areas, the trend in infant mortality for rural areas closely mirrors the pattern for the total sample. Urban areas experienced fairly low levels of infant mortality for the pre-2005 birth cohorts (49 deaths per 1000), which dropped even further for the 2005/06 cohort (27 deaths). However, in urban areas infant mortality increased slightly for the 2007/08 birth cohort to 33 deaths and then stagnated.

Breakdown of the trends in infant mortality by administrative regions reveals much more complex patterns. Between the pre-2005 and 2005/06 birth cohorts, infant mortality declined in all regions except Bongalova, where it increased slightly from 43 to 47 deaths per 1000 live births. Between the 2005/06 and 2007/08 birth cohorts, substantial declines in infant mortality can be observed in several regions (e.g., Ihorombe, Alaotra Mangoro, Boeny, Atsimo Andrefanna, and Sava). But during the same period, infant mortality increased substantially in several other regions (e.g., Haute Matsiatra, Vatovavy Fitovinany, Atsinanana, Analanjirofo, Sofia, and Diana). Similarly, when comparing infant mortality levels for the 2007/08 and 2009/2011 birth cohorts, substantial improvements are observed in many regions (e.g. Itasy, Atsinanana, Diana), but increasing mortality levels in several other regions (e.g., Sofia, Atsimo Andrefana, Sava).

### Trends in ITN ownership

Tables 4 and 5 show trends in the percentage of households that had at least one bed net and that had at least two bed nets, respectively, at the time of each of the four surveys. Results are presented for the total sample, as well as by region and type of place of residence.

**Table 4:** Percentage of households that have at least one bed net, by survey, region, and place of residence (Source: 2008/9 MDHS; 2011 MIS; 2012–13 ENSOMD; 2013 MIS)

	Survey year			
	2008–9 DHS	2011 MIS	2012–13 MDG	2013 MIS
Antananarivo				
Analamanga	33.1	50.4	35.3	38.8
Vakinankaratra	26.4	71.3	19.3	34.6
Itasy	10.1	9.6	9.4	12.7
Bongolava	44.1	97.1	73.5	81.8
Fianarantsoa				
Haute Matsiatra	52.5	47.6	30.6	27.9
Amoron i Mania	24.6	26.9	27.2	14.4
Vatovavy Fitovinany	79.3	97.1	90.4	96.5
Ihorombe	65.7	98.0	71.8	38.6
Atsimo Atsinanana	73.6	98.1	92.3	97.2
Toamasina				
Atsinanana	80.4	94.1	92.6	95.8
Analanjirofo	91.8	99.5	92.0	96.3
Alaotra Mangoro	75.7	95.0	77.3	84.6
Mahajanga				
Boeny	89.6	97.5	77.7	84.0
Sofia	86.7	98.8	88.2	90.0
Betsiboka	76.3	99.9	82.4	95.0
Melaky	78.4	100.0	84.5	77.0
Toliary				
Atsimo Andrefana	75.1	95.0	62.2	43.4
Androy	73.5	98.0	65.3	63.7
Anosy	78.0	88.2	71.6	97.1
Menabe	79.0	97.0	87.6	82.9
Antsiranana				
Diana	91.4	95.3	92.3	85.0
Sava	89.4	97.7	89.8	97.3
Residence				
Rural	65.9	82.2	64.4	70.6
Urban	78.6	91.2	76.1	85.4
Total	66.3	83.1	65.9	72.0

**Table 5:** Percentage of households that have at least two bed nets, by survey, region, and place of residence (Source: 2008/9 MDHS; 2011 MIS; 2012–13 ENSOMD; 2013 MIS)

	Survey year			
	2008–9 DHS	2011 MIS	2012–13 MDG	2013 MIS
Antananarivo				
Analamanga	10.0	24.9	14.6	16.9
Vakinankaratra	7.4	32.6	7.8	15.2
Itasy	2.6	3.5	2.0	1.8
Bongolava	6.1	68.7	35.2	41.7
Fianarantsoa				
Haute Matsiatra	16.6	24.1	12.4	11.9
Amoron i Mania	8.1	18.2	13.4	6.7
Vatovavy Fitovinany	29.6	70.3	64.1	69.4
Ihorombe	17.5	71.8	35.9	10.9
Atsimo Atsinanana	27.6	68.1	53.1	61.0
Toamasina				
Atsinanana	38.9	60.4	59.7	64.5
Analanjirofo	49.0	76.4	53.3	50.0
Alaotra Mangoro	27.4	70.8	44.2	48.8
Mahajanga				
Boeny	52.9	74.2	42.4	43.0
Sofia	40.1	79.8	53.4	47.8
Betsiboka	32.7	74.6	50.2	51.4
Melaky	37.1	72.4	48.1	39.6
Toliary				
Atsimo Andrefana	25.5	45.0	19.3	11.3
Androy	25.6	61.7	25.2	18.1
Anosy	32.3	40.0	31.7	54.6
Menabe	34.1	55.0	41.1	30.7
Antsiranana				
Diana	48.0	54.1	49.9	43.0
Sava	41.7	55.4	52.6	61.9
Residence				
Rural	25.8	51.8	33.8	37.9
Urban	44.0	60.5	46.2	50.7
Total	27.7	52.6	35.4	39.1

The results presented in Table 4 show that in 2008/09, 66 % of households owned at least one net. By 2011, shortly after the 2009 and 2010 mass distribution campaigns, 83 % of households reported having at least one bed net. The percentage having at least one bed net dropped to 66 % by 2012/13 and then slightly increased to 72 % in 2013. Breakdown by rural/urban residence shows that urban households are more likely than rural households to have at least one bed net, but the trends in both rural and urban areas follow a similar pattern.

Analysis by administrative region shows that following the 2009 and 2010 distribution campaigns the percentage of households that own at least one bed net increased in all regions except Itsay (10.1 % in 2008/9 to 9.6 % in 2011), Haute Matsiatra (52.5 to 47.6 %), and Amoron Mania (24.6 to 26.9 %). However, by 2012/13 the percentage of households that own one or more bed nets had declined in most regions. Major declines of more than 20 percentage points were observed in Vakinankaratra (71.3 to 19.3 %), Bongolave (97.1 to 73.5 %), Ihorombe (98.0 to 71.8 %), Atsimo Andrefana (95.0 to 62.2 %), and Androy (98.0 to 65.3 %). Declines in most other regions were smaller, but nevertheless substantial. There were only four regions where the percentage of households that own at least one net remained roughly constant between 2011 and 2012/13 (Itasy, Amoron i Mania, Atsinana, and Diana).

Between the 2012/13 and 2013 surveys, the percentage of households who own at least one net increased in most regions. Nevertheless, substantial declines were observed in Amoron i Mania (27.2 to 14.4 %), Ihorombe (71.8 to 38.6 %), Melaky (84.5 to 77.0 %), Atsimo Andrefana (62.2 to 43.4 %), and Diana (92.3 to 85.0 %).

As anticipated, the percentage of households owning at least one bed net increased between 2012/13 and 2013 in all six regions targeted by the 2012 mass distribution campaign (Vatovavy Fitovinany, Atsimo Atsinanana, Atsinanana, Analanjirofo, Anosy, and Sava).

Table 5 shows trends in the percentage of households that own two or more bed nets. For the total sample, this percentage increases from 27.7 % in 2008/09 to 52.6 % in 2011, and then drops to 35.4 % in 2012/13. By 2013, the percentage had slightly increased again, to 39.1 %. Breakdown by type of place of residence shows that the trends in rural and urban areas follows a similar pattern. Urban households are substantially more likely than rural households to own two more nets. The gap between rural and urban areas diminished considerably following the 2009 and 2010 mass distribution campaigns, but has since widened again.

Regional analysis shows that in most regions, the percentage of households that own two or more ITNs increased substantially between 2008/09 and 2011, but then experienced a large decline by 2012/13. A notable exception to this pattern is Itasy, where the percentage of households that own two or more bed nets remained below 4 % in all four surveys.

Between 2012/13 and 2013, the changes in the percentage of households that own at least two bed nets vary more by region. The percentage of households that own two or more bed nets increased in all six regions targeted by the 2012 mass distribution campaign (Vatovavy Fitovinany, Atsimo Atsinanana, Atsinanana, Analanjirofo, Anosy, and Sava). However, notable increases in the percentage of households owning two or more bed nets were also observed in Vakinankaratra (7.8 to 15.2 %) and Bongolova (35.2 to 41.7 %). By contrast, between the 2012/13 and 2013 surveys the percentage of households owning two or more bed nets decreased considerably in Ihorombe (35.9 to 10.9 %), Melaky (48.1 to 39.6 %), and Menabe (41.1 to 30.7 %).

### Association between ITN ownership and infant and child mortality

The results of the Cox proportional hazard models estimating the association between the predictor variables and child mortality are shown in Table 6. The first model only includes the calendar year, without other control variables. The results show that compared with previous years (2003–08), the risk of dying was 22 % lower in 2009 (p < .10), and 35 % in 2010 (p < .01). However, the risk of dying subsequently increased and by 2011 and 2012/13 the risk of dying had reverted to the levels that had been observed in 2003–08. The second model shows whether living in a household that owns at least one ITN affects the risk of dying. After controlling for the secular trend in mortality and other control variables, children who live in a household that owns at least one ITN are 22 % less likely to die than other children. However, the hazard ratios for the time trend remain virtually unchanged, which suggests that increasing bed net ownership cannot fully explain the observed trends in child mortality. It is also observed that the risk of dying is significantly higher during the malaria season, despite the fact that the model controls for ownership of at least one ITN. In other words, ownership of one ITN in the household is not sufficient to eliminate the risk. Other factors that significantly affect child mortality include mother’s age and parity, socio-economic status, and cluster-level diarrhoea prevalence. It is unclear why children born to high SES mothers have an elevated mortality risk.

**Table 6:** Adjusted hazard ratios from Cox proportional hazard models on all-cause child mortality (under age 5)

	All regions			High P*f*PR_2–10_ regions		
	(1)	(2)	(3)	(4)	(5)	(6)
Calendar year						
<2009 (ref)	1.00	1.00	1.00	1.00	1.00	1.00
2009	.78*	.76**	.77*	.77	.72*	.74*
2010			.67***	.88	.83	.86
2011	.65***	.65***	1.08	1.12	1.09	1.11
2012–13	1.03	1.06	1.18	1.34*	1.34*	1.37
	1.08	1.16				
Household owns at least one net		.78**	–		.60***	–
Household owns at least two nets		–	.76***		–	.69***
Malaria season		1.21**	1.20**		1.16	1.16
P*f*PR_2–10_ level (%)						
10–19		1.00	1.00		–	–
20–29		1.09	.96		–	–
30–39		1.21	1.14		–	–
40+		1.20	1.16		–	–
Mother’s age					.97***	.97***
Parity		.97***	.97***			
		1.19***	1.19***		1.19***	1.20***
Mother’s level of education						
None		1.00	1.00		1.00	1.00
Primary		1.02	1.01		.95	.92
Secondary		1.02	1.03		1.19	1.18
Higher		.74	.74		1.50	1.56
Wealth quintile						
First (poorest)		1.00	1.00		1.00	1.00
Second		1.01	1.00		.88	.88
Third		1.15	1.16		1.11	1.10
Fourth		1.21	1.20		1.03	1.01
Fifth (wealthiest)		1.64***	1.60***		2.16***	2.05***
Urban residence		.82	.83		.64**	.68**
Female child		.94	.94		.93	.94
dpt3 prevalence (cluster)		1.00	1.04		.89	.92
Diarrhea prevalence (cluster)		5.30***	5.45***		4.56***	4.88***

Model 1 shows the unadjusted hazard ratios by survey year for all regions, while models 2 and 3 show the adjusted hazard ratios. Model 4 shows the unadjusted hazard ratios by survey year for high P_f_PR_2–10_ regions only, while Models 5 and 6 show the adjusted hazard ratios

*** p < .01

** p < .05

* p < .10

Model 3 in Table 6 shows a similar analysis of the effect of having two or more (rather than one or more) ITNs in the household, compared to having one ITN or no ITNs. The results are nearly identical as those for model 2. It is noteworthy that having two or more bed nets in the household is still not sufficient to eliminate the increased risk of dying during the malaria season.

Because the effect of the bed net mass distribution programmes are most likely to affect child mortality in regions where malaria is a severe problem, these analyses were repeated for regions with a *Pf*PR_2–10_ exceeding 40 % (Vatovavy Fitovinany, Atsimo Atsinanana, Atsinanana, Analanjirofo, Boeny, Sofia, Betsiboka, Melaky, Menabe, Diana, and Sava). The results are shown in models 4–6. Although the secular trend in these high transmission regions is similar to what was observed for the total sample, there are no significant differences in child mortality between 2003/08 and 2011. The data further suggest that during calendar years 2012–13 the risk of dying may have been higher than in 2003–08 (p < .10). Model 5 shows that in high transmission regions ownership of at least one ITN in the household is associated with a 40 % reduction in child mortality (p < .01), after controlling for other factors. In these high transmission regions, the risk of dying does not vary by season. This is common in areas where malaria is highly endemic and that do not have a very pronounced seasonal pattern. Model 6 indicates that ownership of two or more ITNs is associated with a 31 % reduction in child mortality (p < .01).

Table 7 shows the same analyses for infant mortality. The first model once again shows that infant mortality risks significantly decreased between 2003/08 and 2010. Compared to the period 2003–08, the risk of an infant dying was 22 % less in 2009 (p < .10) and 30 % less in 2010 (p < .05), but higher in later years. The second model shows that ownership of at least one bed net is associated with a 22 % reduction in infant mortality, after controlling for other factors (p < .05). Mother’s age and parity, socioeconomic status and cluster-level diarrhea prevalence also significantly affect infant mortality. It is noted that after controlling for bed net ownership and other factors, the risk of an infant dying does not significantly vary by season. Model 3 repeats the analysis using household ownership of two or more ITNs as the treatment variable. After controlling for other factors, ownership of two or more bed nets is associated with a 19 % reduction in infant mortality. Once again, after controlling for ownership of two or more bed nets and other factors, the risk of an infant dying does not significantly increase during the malaria season.

**Table 7:** Adjusted hazard ratios from Cox proportional hazard models on all-cause infant mortality (under age 1)

	All regions			High P*f*PR_2–10_ regions		
	(1)	(2)	(3)	(4)	(5)	(6)
Calendar year						
<2009 (ref)	1.00	1.00	1.00	1.00	1.00	1.00
2009	.78*	.76**	.76*	.83	.79	.79
2010		.71**	.72**	.94	.89	.91
2011	.70**	1.31**	1.33**	1.36*	1.31	1.33
2012–13	1.26*	1.24	1.25*	1.36*	1.35	1.36*
	1.15					
Household owns at least one net		.78**	–		.66**	–
Household owns at least two nets		–	.81**		–	.79**
Malaria season		1.11	1.11		1.03	1.03
P*f*PR_2–10_ level (%)						
10–19		1.00	1.00		–	–
20–29		1.02	.96		–	–
30–39		1.28*	1.20		–	–
40+		1.20	1.14		–	–
Mother’s age		.97***	.97***		.97***	.97***
Parity		1.18***	1.19***		1.18***	1.18***
Mother’s level of education						
None		1.00	1.00		1.00	1.00
Primary		1.02	1.01		.94	.92
Secondary		1.10	1.10		1.30	1.29
Higher		.88	.88		1.92	1.92
Wealth quintile						
First (poorest)		1.00	1.00		1.00	1.00
Second		1.06	1.05		.88	.87
Third		1.24*	1.24*		1.18	1.16
Fourth		1.23	1.22		1.06	1.05
Fifth (wealthiest)		1.66***	1.61***		2.17***	2.08***
Urban residence		.86	.87		.63**	.65**
Female child		.92	.93		.92	.93
dpt3 prevalence (cluster)		1.19	1.15		1.00	1.02
Diarrhea prevalence (cluster)		5.17***	5.3***		3.78***	3.94***

Model 1 shows the unadjusted hazard ratios by survey year for all regions, while models 2 and 3 show the adjusted hazard ratios. Model 4 shows the unadjusted hazard ratios by survey year for high P_f_PR_2–10_ regions only, while Models 5 and 6 show the adjusted hazard ratios

*** p < .01

** p < .05

* p < .10

When the analysis is restricted to high transmission areas, it is observed that there were no significant changes in infant mortality for the period from 2003/09 to 2010. However, in subsequently years infant mortality increased. In both 2011 and 2012/13 the risk of an infant dying was 36 % higher than in 2003/08 (see model 4). Model 5 shows that in high transmission areas, ownership of at least one bed net is associated with a 34 % reduction in infant mortality (p < .05), and after controls for bed net ownership and other factors the risk of dying does not significantly increase during the malaria season. It is noteworthy that in these high transmission areas, infants living in rural areas have significantly higher mortality than those in urban areas, even after controlling for ITN ownership. As shown in model 6, in these high transmission regions ownership of two or more ITNs is associated with a 21 % net reduction in infant mortality, compared to children living in households that have only one net, or no nets at all.

## Discussion

Malaria is one of the major public health problems that face Madagascar. To reduce the malaria burden, the Government of Madagascar, with support from international aid agencies, is implementing a major prevention strategy that includes mass distribution of insecticide-treated bed nets. The goal is to provide all households with at least one insecticide-treated net for every two persons. The aim of this paper is to examine the association between ITN ownership and infant and child mortality.

The results of the lifetable analyses show that there was a rapid decline in infant and child mortality, which was followed by near stagnant mortality levels. However, this period of apparently stagnant infant and child mortality levels at the national level resulted from averaging regional level mortality trends. At the regional level, there was significant variation in infant and child mortality trends. The results show that while infant and child mortality levels improved in some regions, they clearly deteriorated in other regions. It is unclear whether these deteriorations are associated with the decline in foreign aid that occurred after the 2009 coup d’état. The suspension of US Government aid to the Government of Madagascar that followed the coup d’état of March 2009 affected support for the health sector [13, 24]. Although funds were reprogrammed to international and local non-governmental organizations, this reduced health sector support may have slowed down the decline in infant and child mortality from 2010 onward, and may have reversed it in areas that international and local non-governmental organizations did not focus on.

Data on household-level bed net ownership confirm that the percentage of households that own one or more bed nets increased substantially following the 2009 and 2010 mass LLIN distribution campaigns. Although the percentage of households that own at least one net reached as high as 81 % by 2011, these coverage levels were not sustained, dropping to 66 % by 2012/13. This decline occurred in nearly all regions. By 2013, the percentage increased to 72 %. Interestingly, this most recent increase in ITN ownership was observed not only in the six regions targeted by the 2012 mass distribution campaign, but also in a number of other regions. Nevertheless, a few regions experienced substantial declines the percentage of households that own one or more bed nets. Analysis of the percentage of households that own two or more nets also show major increases after the 2009 and 2010 mass distribution campaigns, followed by a substantial decline. The finding that mass distribution campaigns appear to have a fairly short-term effect on net ownership highlights the importance of supplementary social marketing and routine LLIN distribution programmes.

A number of previous studies have found that bed net ownership is associated with a reduction in all-cause mortality [26, 27]. For example, a Cochrane review of individual and cluster randomized trials on the protective efficacy of ITNs found that ITN ownership is associated with an 18 % reduction in child mortality [26]. A more recent meta-analysis found that household ownership of at least one ITN was associated with a 23 % reduction in mortality between aged 1 month and age five, while a study of Malawi found that household ownership of at least one ITN was associated with a 25 % reduction in all-cause child mortality [22, 28]. Although the analyses assess the associations between bed net ownership and all-cause child mortality under programmatic conditions, rather than as part of a controlled trial, the results from this study are consistent with these earlier findings. The analysis suggests that in Madagascar household ownership of at least one bed net is associated with a 22 % net reduction in all-cause child mortality as well as all-cause infant mortality. Ownership of two or more bed nets (compared to having none or only one) was associated with similar reductions in the risk of infant and child mortality. In high transmission areas, bed net ownership was associated with even larger reductions in mortality.

This analysis has several limitations, with the major limitation being that measures of bed net ownership are current status variables that refer to ownership at the time of the survey, which may not correspond with ownership at the time when the children were at risk. Third, while the analysis takes several confounding factors into account that may influence infant and child mortality, it is likely that there are other confounding factors that were not analysed here and that may explain the unexpected positive hazard ratios for children from high SES households. Additionally, though the analysis controlled for regional level malaria risk, it did not include direct measures of climactic variability in this analysis because not all survey data could be linked to locations below the regional level. There is a possibility that some changes in infant and child mortality are due to fluctuations in malaria risk due to short-term local climate variability which this analysis did not account.

Future research may be able to refine the estimates of the extent to which ITN ownership is associated with a reduction in infant and child mortality in Madagascar. Suggested refinements include using a time-varying covariate for ITN ownership (estimated based on the date each ITN in the household was first obtained) and more advanced procedures to eliminate the effect of potential confounding factors. The latter may include using propensity score matching to create equivalent control and treatment groups that are equivalent on a large number of variables, and adding controls for district-level covariates and time-varying environmental covariates such as temperature and rainfall.

## Conclusions

Data on household-level bed net ownership confirm that the percentage of households that own one or more bed nets increased substantially following the 2009 and 2010 mass LLIN distribution campaigns. Additionally, all-cause child mortality in Madagascar has declined over the period 2008–2013. Bed net ownership was associated with a 22 % reduction in all-cause child mortality hazard in Madagascar. Mass bed net distributions contributed strongly to the overall decline in child mortality in Madagascar over the period 2008–2013. However, the decline was not solely attributable to increases in bed net coverage, and nets alone were not able to eliminate the majority of child mortality hazard across the island.

## Footnotes

1 Note that the public use data files for the MDG survey have not yet been released; the present analysis is based on a preliminary version of the dataset provided to us by INSTAT.

2 Note that the 2012/13 MDG survey did not collect altitude data.

3 The 2011 and 2013 MIS surveys also contain an abbreviated birth history for children born during the 6 years prior to the survey. However, while these surveys enquired whether the child is still alive, they do not include the date of death nor the age at death for deceased children.
